# ‘We are planning to leave, all of us’—a realist study of mechanisms explaining healthcare employee turnover in rural Ethiopia

**DOI:** 10.1186/s12960-018-0301-0

**Published:** 2018-08-13

**Authors:** Joris van de Klundert, Judith van Dongen- van den Broek, Ebrahim Mohammed Yesuf, Jasmijn Vreugdenhil, Saeid Mohammed Yimer

**Affiliations:** 1 Erasmus School for Health Policy & Management, Erasmus University Rotterdam, Rotterdam, The Netherlands; 2 Prince Mohammad Bin Salman College, King Abdullah Economic City, Saudi Arabia; 3 Transvorm, Tilburg, The Netherlands; 4 Department of Public Health, College of Health Science, Samara University, Semera, Ethiopia; 5 Department of Management, College of Business and Economics, Samara University, Semera, Ethiopia

**Keywords:** Human resource management, Retention, Turnover intentions, Ethiopia

## Abstract

**Background:**

We study healthcare employees’ turnover intentions in the Afar National Regional State of Ethiopia. This rural region is experiencing the globally felt crisis in human resources, which is inhibiting its ability to meet health-related sustainable development goals.

**Methods:**

Realist case study which combines literature study and qualitative analysis of interview and focus group discussion data, following a realist case study protocol.

**Results:**

A large majority of employees has turnover intentions. Building on Herzberg’s two-factor theory, person-environment fit theory, as well as recent sub-Saharan evidence, analysis of the collected data yields four turnover mechanisms: (1) lack of social and personal opportunities in the region, (2) dissonance between management logic and professional logic, (3) standards of service operations are hard to accept, and (4) lack of financial improvement opportunities.

**Conclusions:**

While the first and fourth mechanisms may be out of reach for local (human resource) management interventions, the second and third mechanisms proposed to explain health workforce turnover appear to be amenable to local (human resource) management interventions to strengthen healthcare. These mechanisms are likely to play a role in other remote sub-Saharan regions as well.

## Background

In recent years, the shortage of healthcare employees working in primary health service delivery processes (henceforth referred to as healthcare employees) has reached such proportions that some even speak of a crisis in human resources for health [1]. While sub-Saharan Africa carries over 24% of the global burden of disease, only 3% of the global health workforce works in this region. The resulting gap between supply (in terms of healthcare workforce) and demand (for health services) results in inability to give proper attention to each client and provide care as needed [1, 2]. Sub-Saharan countries struggle to cope with these negative consequences and to deliver minimum standards of healthcare [1, 3]. This threatens the ability to accomplish health-related Sustainable Development Goals, particularly in ensuring healthy lives and promoting well-being for all [4]. Each further decrease in the number of healthcare employees brings further negative health consequences [3]. Senior officials from countries such as Ethiopia consider the shortage of healthcare employees to be the main obstacle to meeting the health challenges [5].

The Federal Democratic Republic of Ethiopia is a developing sub-Saharan country, ranked 173 out of 186 on the Human Development Index [6] and 127 out of 148 on the Global Competitiveness Index [7]. Bearing in mind the poverty and history of conflict in the country, it is not surprising that Ethiopia is struggling with the provision of healthcare. In 2010, there were 66 314 healthcare workers in Ethiopia, including health extension workers, about 0.8 per 1 000 inhabitants, and less than 0.5 per 1 000 in rural areas where 85% of the Ethiopian population lives [8]. In addition, the reported tendency among healthcare employees to move from rural to urban areas structurally threatens healthcare availability for the rural population [1].

Compared to other rural regions in sub-Saharan Africa and Ethiopia, the Afar National Regional State (henceforth Afar Region) is among the least developed and has chronic shortages of healthcare professionals which negatively impact population health. The area consists mainly of desert and has a harsh climate with temperatures as high as 50 °C in summer [9]. Historically, the Afar Region is known to be unstable with many brooding conflicts and violent outbursts for such reasons as nationalism, inter-communal conflict, political conflict between various political parties, and sometimes conflict over resources between clans [10].

While current national and provincial policies actively direct health professionals towards the poorer areas such as the Afar Region, the numbers of health professionals continue to be reduced by staff turnover—employees leaving the organisation [11]. As staff turnover inhibits the effectiveness of any solution approach, it is essential to understand the causes of staff turnover and develop corresponding countermeasures.

Several studies on factors associated with healthcare employee turnover in African counties have been conducted [12–16]. As factors associated with actual turnover are often difficult to study, these (and other) studies consider factors associated with turnover intentions. Turnover intentions are defined as a conscious wilfulness to look for a job in other organisations [17]. Both theory and empirical evidence support the use of turnover intentions as a proxy for turnover [18, 19]. We note, however, that contextual factors might influence whether turnover intentions lead to actual turnover [20].

Contextual factors such as organisational and environmental factors have recently also become more prominent in studies on turnover intentions, both in developing and developed countries [20]. The degree of person-environment fit and the type of human research practices in place are examples of such factors [18, 19, 21]. These recent developments add to previous studies which focused on demographics, attitudes and behaviour of individual employees, such as job satisfaction and commitment, in explaining turnover intentions and actual turnover in healthcare [20].

Various authors stress the importance of the local cultural, political and socio-economic context in determining factors affecting turnover intentions [2, 22]. Nevertheless, remote, rural, sub-Saharan areas such as the Afar Region which experience such severe negative consequences of staff turnover are underrepresented in scientific literature on this topic. Moreover, these studies often use quantitative surveys of general factors associated with turnover intentions of which the validity in the studied context remains unclear; some factors may not be valid, other valid factors may be missing out. Moreover, to develop countermeasures, it does not suffice to identify factors. It requires to reveal the mechanisms through which contextual factors influence turnover intentions, as will be our research aim.

Given the nature of our research aim, we adopt a realist paradigm and consider context-mechanism-outcome configurations [23]. Specifically, we set out to find the mechanisms that explain how the context of the health service system relates to the outcome of healthcare employee turnover intention. To this purpose, we conduct an in-depth observational case study (as in [24]), with the following research question: *Which mechanisms underlie staff turnover intention in the Afar Region in Ethiopia?* In line with the research aim, the study focussed on healthcare employees who are directly involved in health service delivery, such as nurses and doctors. The realist approach results in middle range theory, which facilitates a more generable understanding of healthcare workforce turnover in remote rural sub-Saharan contexts (as explicitly addressed in the ‘Discussion’ section).

## Methods

Using the conceptual framework of the context-mechanism-outcome configuration [23], our methods aimed to identify mechanisms that are likely to produce turnover intention among healthcare employees at work in the Afar Region health system. Taking a realist case study approach, we aimed to derive these mechanisms through the following steps, which are adapted from the framework for realist evaluation [23, 24]:

1. Formalise theories to be tested regarding the context-mechanism-outcome configurations. To this purpose, we formulated a set of initial hypotheses based on general theories and evidence on turnover in sub-Saharan Africa. These hypotheses represent initial mid-range theories on mechanisms that explain how (the outcome) turnover is affected by (the intervention) HR practices in (the context of) the Afar Region (see Table 1).
2. Collect data to investigate the hypotheses. To enable a broad, generative analysis, data collection was designed to cover the proposed theoretical frameworks more broadly than through the initial hypotheses and to elicit further hypotheses from the responses.
3. Test the initial hypotheses and potential follow-up hypotheses using the collected data.
4. Assessment and interpretation of the analysis to verify whether the theories and hypotheses are supported or refuted. An explicit report of the assessment and interpretation is given in the ‘Results’ section.
5. Generalisation of the findings to other sub-Saharan contexts is covered in the ‘Discussion’ section.

**Table 1:** Initial hypothesis on mechanisms of turnover intentions

Hypothesis number	Hypothesis	Theoretical support
Hypothesis 1 (H1)	Poor salary, career opportunities and management support reduce motivation and job satisfaction and push employees away from the public health service organisations in the Afar Region	Two-factor theory
Hypothesis 2 (H2)	Employee perceptions that alternative health service organisations offer better job challenges, responsibility and autonomy pull employees towards these alternatives	Two-factor theory
Hypothesis 3 (H3)	Employees are pushed away by lack of personal fit with their employer, in terms of job rewards: salary	Person-environment fit
Hypothesis 4 (H4)	Employees are pushed away by lack of personal fit between their professional logic and the dominant organisational logic in the employer organisation, especially with regard to priority and autonomy for quality of care	Person-environment fit
Hypothesis 5 (H5)	Employees are pulled towards alternative organisations if they think their professional logic is a better match with the dominant organisational logic of the alternative organisation (e.g. with regard to priority and autonomy for quality of care)	Person-environment fit

## Results

### Step 1: Formalising theories

#### Push and pull factors

The literature on employee turnover intentions in sub-Saharan Africa mentions various push and pull factors of turnover intentions [25, 26]. Push factors or features relate to the current workplace, region or country and cause employees to seek alternatives. Pull factors relate to alternative employment opportunities that draw employees towards other organisations, regions or countries. Push and pull factors are known to be interrelated, as explained further below [4]. First, let us review the evidence of the dominant push and pull factors obtained in sub-Saharan Africa.

#### Evidence: push factors

Dissatisfaction with salary is reported to be the number one reason for Ethiopian healthcare employees to quit their first job [27]. Specifically, 40 to 45% of the doctors interviewed indicated low salary as the most important reason. Moreover, quitting their first job actually paid off for almost 50% of the doctors, who received a higher salary (ibid.). Salary is also a major factor in healthcare employee turnover in four other sub-Saharan African countries [26] and confirmed as an important push factor [28, 29]. Low salary is mentioned as a common reason to move from public to private healthcare [26, 30]. Doctors switching from public to private healthcare to increase their income are reported to be particularly frequent in rural areas where it forms a main reason for the shortage of health human resources [26].

Next to (low) salary, there is evidence that (poor) working conditions are an important push factor for sub-Saharan health workers. The following dimensions of working conditions have been identified as push factors: physical conditions of the work place, location of the workplace, personal safety and health, workload and management support.

Doctors and nurses consider the physical condition of the workplace as one of the most important job characteristics related to job satisfaction [27]. For example, lack of equipment and drugs may prevent employees from achieving their goal of providing care of good quality [29], and thereby reduce their job satisfaction. The physical condition of a workplace is often related to its location. In rural areas, more healthcare employees are dissatisfied with the physical condition of their workplace than in urban areas [27].

This brings us to another dimension of working conditions: the location of the workplace. The trend among healthcare employees is to migrate from rural to urban areas and from developing to developed countries [1]. Healthcare employees migrate to urban areas to find better career opportunities, both at the current employer and at alternative organisations. As a result, healthcare employees are underrepresented in rural areas in both the public and the private sector [27].

Personal safety and health are a third dimension of working conditions. Personal insecurity is increasingly pushing away healthcare employees from their workplace. Lack of safety can occur within the healthcare organisation but also in the external environment [26]. Conflict and war are among the most important reasons for healthcare employees to migrate. Accordingly, countries with a history of conflict tend to have a smaller healthcare workforce [29]. Furthermore, violence may also occur at the workplace and push employees out [28].

Workload forms another dimension of working conditions and the HIV/AIDS epidemic has increased the workload [29]. First, because it has increased demand for health services. Secondly, healthcare workers can get infected with HIV/AIDS as well, meaning that they will have to leave their jobs and thus increase the workload of the remaining staff. Workload may also play a role in the form of emotional burden on employees working, for instance, with terminally ill patients, which may cause healthcare workers to quit their jobs [29]. Workload effects may multiply as every departing employee increases the workload of the remaining staff [4]. A fifth and final dimension is management support. Several authors mention lack of support from supervisors as a push factor [26, 29]. Sixty percent of Ethiopian healthcare employees experience job dissatisfaction because of lack of management support [27].

#### Evidence: pull factors

Learning opportunities in other countries to improve skills and to grow intellectually are pulling healthcare employees abroad [28]. This covers not only education and refresher courses. Employees can also be pulled by the opportunities offered by facilities, such as internet access and modern libraries. Several other authors confirm learning opportunities to be a pull factor in the sub-Saharan region [3, 30]. Nurses actively seek learning experiences as they are encouraged to develop personally. Career advancement thus forms a second pull factor stimulating migration [26].

There are more healthcare jobs in developed countries than in developing countries. The ageing of society in developed countries is leading to fewer new healthcare employees and increasing demand for healthcare employees, potentially resulting in structural shortages [1]. The vacancies cause developed countries to pull healthcare professionals from other countries, e.g. by assisting with immigration [28]. Moreover, developed countries proactively, or aggressively, recruit healthcare workers [25, 28, 30] (i.e. ‘active encouragement of healthcare employees to leave developing countries to be employed in a developed country’), which is particularly mentioned as a pull factor for the sub-Saharan region [4, 26, 31]. This pull factor may in turn lead to (aggressive) recruitment by developing countries to compensate for the resulting migration of domestic healthcare employees [30].

#### Two-factor theory

The concrete push and pull factors listed above can be understood from theoretical perspectives using more general constructs to explain employee turnover. Evidence suggests that poor motivation and dissatisfaction are the most important predictors of turnover intentions and actual turnover in low- and middle-income countries [13]. Two-factor theory distinguishes motivational factors from hygiene factors [32]. Hygiene factors are extrinsic to the employee and cause dissatisfaction and lower motivation when absent. The job context-related push factors such as salary and supervisory support are hygiene factors [33, 34]. They relate to lower-order needs of employees from the viewpoint of Maslow’s hierarchy of needs [35]. When lower-level needs are secured, intrinsic, motivational factors relate to the fulfilment of higher-level needs [34, 36]. The pull factors ‘career advancement’ and ‘education opportunities’ relate to such higher-level needs and have been shown to be predictors of turnover intentions for nurses [34, 37].

#### Person-environment fit theory

Person-environment fit theory to explain organisational behaviour has also served as a framework to understand push factors [38, 39]. This theory posits that a poor fit, or mismatch, between the individual employee and their environment may increase turnover intentions, while a good fit is likely to result in such positive outcomes as job satisfaction and motivation, which promote retention. Examples of individual characteristics are needs, abilities, norms and values. Environmental dimensions include job rewards, job demands, cultural values and group characteristics. As a result, different types of person-environment fit can be identified, such as the fit between the needs of the individual and the rewards of the organisations, in relation to salary as a push factor.

In healthcare, professional identity has been identified as a personal dimension of particular relevance to the fit with the institutional environment. Healthcare professionals (e.g. doctors and nurses) often adopt a professional logic which does not fit the organisational logic, i.e. the belief systems in the organisational environment. For example, healthcare professionals value professional autonomy and give priority to quality of care over financial objectives, which is not always respected by management or reflected in organisational processes [40–44]. Lack of autonomy and respect causes professionals to perceive a misfit between themselves and their work environment, thus forming a push factor.

Conversely, one might also conjecture that perceptions about improvement in person-environment fit for other organisations/regions/countries function as a pull factor. This conversion forms an example of the interrelationship between push and pull factors: poor person-environment fit is a push factor, perceived good fit elsewhere a pull factor. Contrary to the person-environment fit theory, the two-factor theory provides no support for such interrelationships, as it relates push and pull factors to different levels in Maslow’s hierarchy of needs.

From the evidence and theory provided above, we propose five hypotheses (see Table 1) to specify the initial programme theory on healthcare employee turnover in the Afar Region.

### Step 2: Data collection

In March 2014, we collected qualitative data (step 2 of the framework) in semi-structured interviews with individuals as well as groups of respondents, according to feasibility and availability. To avoid being misguided by the initial hypotheses, the interviews were not explicitly structured around the hypotheses, but rather around the push and pull factors. We asked openly for push and pull factors and covered the push and pull factors known from literature. For each of the factors mentioned or confirmed by the respondent(s), we asked for an assessment of relevance and for explanations of how they worked to elicit the underlying mechanisms.

The employees were interviewed in three focus groups, two of which were single sex groups. In addition, we interviewed four managers and four staff members individually. Table 2 describes the respondents. Respondents were purposefully selected to form a varied, relevant and representative sample of the local health system, and we stopped selecting additional respondents when we reached saturation. Some respondents worked in Semera, the capital of the Afar Region; others in remoter areas. For safety reasons and travel constraints, the interviews did not cover all districts of the Afar Region. All respondents were from Administrative Zones 1, 2 and 4. The most Northern part of Zones 2 and Zones 3 and 5 was excluded. Interviews were conducted mostly in English. When required, the local researchers involved provided translation. All interviews were recorded with consent of the participants and transcribed.

**Table 2:** Overview of key informants and participants in focus group discussions

Respondent(s)	Gender	Age	Manager/Employee	Occupation(s)
*FGD 1*	4 x M	29.25 (avg)	E	Nurses
*FGD 2*	6 x F	26.8 (avg)	E	5 nurses, 1 medical doctor
*FGD 3*	4 x M 2 x F	24.5 (avg)	E	3 nurses, 1 medical doctor, 2 laboratory technicians
*Key informant 1*	M	40	E	District’s health office employee for prevention
*Key informant 2*	M	29	M	Head of a governmental health bureau
*Key informant 3*	M	29	E	Employee at the health bureau
*Key informant 4*	M	42	E	Employee at the Semera and Dubti local health offices
*Key informant 5*	M	27	E	Laboratory technician
*Key informant 6*	M	38	M	Project coordinator at an NGO
*Key informant 7*	M	41	M	In-share owner of a private healthcare clinic
*Key informant 8*	M	32	M	In-share owner of a private healthcare clinic

Table 2 provides an account of focus group discussions and individual interviews, including the gender, age, role and occupation of the respondents. In addition, the type of data collection method (individual interview or focus group) is also indicated.

For reasons of transparency and verification, we now present an analysis of the collected qualitative data, to assess whether the proposed and other factors influenced turnover intentions in the Afar Region. To this purpose, we coded the transcribed interviews according to the factors listed above and labelled the newly proposed ones. We classified factors as dominant if they were mentioned by at least half of the respondents.

#### Turnover and turnover intentions

Respondents unequivocally confirmed that healthcare employee turnover and turnover intentions are common among healthcare professionals in the Afar Region. As turnover results from turnover intentions, we present a quote from the second focus group discussion (FGD 2) illustrating the omnipresence of turnover intention: ‘We are planning to leave, all of us. We have the intention to leave. Most of us don’t want to stay for more than two years.’ Indeed, most employee respondents admitted to thinking about leaving, and all respondents knew at least one direct colleague who had left.

While there is no official data on turnover rates, respondents agree that the rates are high. An employee at a district health office estimated the district turnover rate to be at least 50% in the last year. An employee at the local health offices in Samara mentioned that in the past year, seven co-workers had left. (The health office employed 10 health professionals at the time of study.) Another employee at the local health bureau said ‘I don’t know the exact incidence of turnover but it is a huge amount. Of the people that I know who left, one person left already after a month. Others left after a year. Even at the health bureau they are saying that there is a huge difficulty.’

The turnover mostly results in migration to other Ethiopian regions, especially to bigger urban areas such as the capital Addis Ababa. For example, a laboratory technician in Logia said ‘If someone can get a job in Addis or another city, he will go there to work. 90% of the people who leave go to another region.’ Switching jobs to the private sector was not uncommon, but clearly less frequent. Migration to other countries appeared to be rare.

#### Push factors: salary

Seven of the eight individual respondents and two of the three FGDs (focus group discussions) named salary as a main reason for employee turnover, and it was often the first reason mentioned. The head of the provincial health bureau explained that ‘The primary reason to leave in this area is salary. Employees leave public healthcare for private healthcare. For example, they go to work at the sugar factory, which has its own clinic. The difference in pay is very big. 5000 birr at the sugar factory and only 1400 birr in public healthcare.’ He added ‘The health office has payment issues. Both experts and managers are on the payroll, so most employees complain about this issue.’ Employees not only leave because of the low salary but also because they sometimes do not receive it.

Salary differences exist not only between the private and public sector but also between regions in Ethiopia. The Afar Region paid a top-up to the national salary, a policy now copied by many other regions, thus diminishing the reducing effect it may have had on turnover (intentions). In addition to the regular salary, supplements and other opportunities to complement it are important, for instance in the form of moonlighting as mentioned in FGD2: ‘In addition [...] the salary is a reason to leave, but also there is no access to private healthcare institutions to work overtime.’ (Earlier evidence also suggests that retention is high for employees who ‘moonlight’ at private organisations [30].) If an area lacks this opportunity, employees might want to move to one where they can indeed work overtime at a private health organisation. This phenomenon is reinforced by the frequent and partly unpaid overtime demanded from employees in the public system: ‘The health professionals that still work here have to work sometimes 18 hours per day when they are on night duty. And they won’t get overtime payment. They get paid for the night work but not for six hours.’ The two respondents owning private clinics decided to open their own businesses because the salaries in the public healthcare organisations were too low.

Respondents confirmed the five dimensions of working conditions suggested in the literature: facilities, location of the workplace, management support, personal safety and health, and perceived workload. While the latter two appear to be less dominant, for ease of presentation, we consider them as five separate factors in Table 2 and in the text below.

#### Push factors: facilities

Employees at public healthcare organisations clearly worded their dissatisfaction with the physical conditions. One respondent in FGD stated ‘We need to have some standards. If the standards are not met, I want to move to another organization [...]It is likely that people want to leave for NGOs because of the assumptions that that kind of organisations are a little bit better in everything, accommodation, everything. And from government to private institutions, that’s also common.’

One of the participants of the first focus group, a medical doctor, talked fervently about her daily struggles due to the lack of facilities and materials: ‘Here in this health centre we don’t have enough equipment to help the patients. Because of that, we don’t have the moral or the interest to treat the patients. You learn how to treat patients in school but here it is impossible. You know their problem but you cannot help them. It is really boring and very frustrating. I might cry sometimes. You see patients suffering. [Respondent cries] Sorry. You know the patient’s problem, you don’t have the necessary medication. You don’t have the necessary equipment. Even if you send them to a hospital - Dubti is the nearest hospital - you know they can’t get the necessary treatment there. So you just stay there and stare at them. We have a health centre here, but it’s just a name and a building. It is not interesting to work here, I’m sorry. Sometimes I hate the time that I am here. If I was somewhere else, I could have helped someone with my education and my knowledge. Here, I am just wasting it. It’s not just about the money. This is really not good.’

Another participant confirmed that working in a workplace with poor physical conditions can be mentally draining: ‘Once I had a patient here who was vomiting blood, so her illness was very severe. The way to help her was to refer her to a hospital. But there is no ambulance here to transport patients, so this patient died. These situations also want to make us quit our jobs.’ In addition, respondents mentioned that the poor facilities sometimes prevented them from practising their knowledge and skills: ‘The main issue that turnover is very high is that we are lacking facilities. We don’t have equipment and facilities to get experience. So, we lose the knowledge that we had before.’

The inability to deliver health services may ultimately solicit aggression from patients and their families, thus endangering employee safety and health. Employee safety and health may be further endangered because of facility layout, capacity and use. This can take the form of having to work unsafely with chemicals, blood, or other laboratory services, or lack of hygiene in patient rooms. Moreover, facilities for nurses on night duty are perceived to be inadequate.

#### Push factors: location of the workplace

A focus group respondent compared location of the workplace to salary: ‘Salary is not the reason to migrate from rural to urban areas. It is the infrastructure. In rural areas there is no light, water. There is no access to some facilities, like shopping. That is the reason. It is not the salary; the salary is not that different.’ Workplace location also affects the lives of employees’ families. Respondents mentioned the lack of good schools, and affordability of and access to health services. ‘Even if we are working in a Woreda health office our organization didn’t offer us a free health service.’ Moreover, some respondents mention that life is expensive in the towns of Samara and Loggia.

Respondents consider the Afar Region to be very quiet: ‘This place is very boring. The whole area is boring. There is nothing to do here. Every day you go to work, you go home, you sleep. So everyone will get bored sometime.’ Some respondents explicitly mentioned the lack of recreational facilities. Added to the harsh and extremely hot desert climate of the region, it causes the regional living conditions to be viewed as unattractive. Many respondents mentioned the difficulty of the climate: ‘Only 15% of the people working here are born in the Afar Region. The people who are not born here have trouble with the environment, especially the people from the highlands.’

The culture of the Afar Region was also mentioned in relation to location as a cause of turnover (intention). First, because of the language barriers with patients and colleagues who speak the local language, which the majority of employees does not understand. In addition to the harsh climate, religious and cultural differences cause many employees to develop a limited commitment with the local community, and to be open to returning to their area of origin or moving on.

#### Push factors: management support

When respondents spoke about management support, it was often with dissatisfaction. Some respondents described the lack of management support in general terms ‘The management support is not that much satisfactory. The regional health office sometimes didn’t recognize our problem and especially leaders don’t consider our problems.’ Other respondents gave detailed accounts of dissatisfying practices, as already quoted under learning and career opportunities. Many respondents perceived a lack of interest and sometimes a lack of respect. ‘They had much attention for financial aspects, but not for the work……There is no motivation, recognition.’ Some respondents mentioned to have quit their jobs because of the lack of interest in operational problems. They said that management did not want to enter the laboratory because of the smell, when asked to attend the problems they had due to the lack of supplies.

Other respondents reported a lack of interest or intimacy as a main factor, even up to the point that it might outweigh salary: ‘It is not only about higher salary. Their management system should be good. It does not matter if the salary is high or not. I would not leave my institution. The management system is more important than salary.’ Another respondent added ‘For me, if the management is okay, I cannot leave. If not, then [laughs]. [...] If the management system has problems, then it’s not okay. That would be the main reason for me to leave.’ Managers were reported to have hit employees. Treatment differences between local and non-local employees were perceived as unfair.

#### Push factors: personal safety and health

While the Afar Region has a history of war and conflict, and presently is still not considered to be fully safe, respondents did not mention these aspects in relation to turnover. Violence did not play a role other than already mentioned above. The same holds for personal health.

#### Push factors: perceived workload

While not mentioned that frequently, some respondents mentioned that with every leaving employee the workload increases for the remaining ones, possibly causing other staff to leave their organisation or region in turn (see also [4]). Moreover, the workload was perceived as high: *‘*The health professionals that still work here have to work sometimes 18 hours per day when they are on night duty.’

We have already discussed how perceived workload may become even higher due to the emotionally demanding conditions and experiences. This appears to be further reinforced by the employees experiencing lack of autonomy or control. In fact, some respondents explicitly mentioned lack of autonomy and control to have caused them to quit their jobs in the past.

#### Pull factors

Respondents recognised the proposed pull factors of learning opportunities, career development and aggressive recruitment. Perceptions of better learning opportunities elsewhere have led to turnover to other jobs in the region or within Ethiopia, but not to migration to other countries. With the recent founding of Samara University, which includes a School of Health Sciences, the attractiveness of learning opportunities at alternative employment organisations was expected to lose some importance. Management, however, was perceived to favour local professionals over non-local professionals, who formed by far the majority of the work force. According to one respondent ‘The management was bad because they only wanted to have benefits for themselves and maybe for the native Afar people. For training and education, they wanted to give priority to themselves.’

Likewise, management has been reported to let personal relationships outweigh qualifications when filling positions: ‘Some managers give priority to people they know in assigning jobs. If someone they don’t know applies for the job, they don’t give it to him.’ Added to the early stage of development of public health organisations in Afar, employees perceived these practices as limiting their career development opportunities. Respondents explicitly mentioned that limited educational and career opportunities negatively affected their motivation and job satisfaction.

The migration flow appears not to be affected by active or aggressive recruitment strategies (see also below under salary) neither from within the country nor abroad. However, active recruitment by international organisations coming to the region has occurred on a modest scale and has been effective.

#### Other factors

When asked directly, the respondents did not suggest any other factors. However, the responses above suggest various alternative factors, which we have covered while discussing the factors on the interview topic list. One such factor is culture, which is covered under location. While culture is certainly closely related to geography (see for instance [45]), we propose that it is not a locational geographical factor, but a distinctly independent sociological factor. To illustrate this point, we observe that the harsh desert climate is a push factor which clearly relates to geography, while the problems of living in a ‘boring place’ where locals speak another language, are cultural.

Likewise, the data suggest that the push-factor management support represents two factors. On the one hand, there is the operational management responsibility to manage scarce resources, e.g. salary payment, availability of equipment, occupational hazards. On the other hand, there is human resource management. Respondents asked for recognition, motivation, respect, autonomy, promotion on the basis of competence and so forth. The domains are interrelated, as priority setting in human resources management impacts health service operations and vice versa. Respondents indicated, however, that poor operational conditions can be excused as a push factor, when the management is ‘okay’. ‘Good’ management or ‘okay’ management appears to be associated with recognition, respect, etc., and with alignment of values and norms.

### Step 3: Testing and generating hypotheses

Next, we sorted the coded texts by factor and further analysed the text to verify/falsify our initial hypotheses. We find no support for H2 and H3 and very limited support for H5. The test of H1 provide mixed results, as H1 in fact embodies six hypotheses, one for each combination of salary, career opportunities and supervisory support on the one hand, and with satisfaction and motivation on the other. In its present general form, H1 is not supported by the data. Our analysis does provide overwhelming support for H4, and we further analyse and interpret it below.

After this first round, we iteratively reconsidered our findings from the literature review and collected data to generate new hypotheses. In each of these iterations, the generated hypotheses were either accepted or rejected, in which case we looked for alternatives. The process ended when none of the newly generated hypotheses were rejected. This resulted in four mechanisms. The data support for these hypotheses is provided in Table 3, which gives the factors and findings from the qualitative analysis in step 2 in columns two and four, and the corresponding mechanisms are presented in (rightmost) column five.

**Table 3:** Summary of interview and FGD findings

	Factor	Relevance	Detailed workings	Mechanisms
Pull	Learning opportunities	Dominant	-Founding Semera University reduces turnover to other regions because of learning opportunities	P-RE-M
			-While most employees come from other regions, local employees/management are favoured for education programmes	Dis-Log
			-Employees unlearn; lose skills because of poor facilities	SOP
	Career opportunities		-Employees perceive big Ethiopian cities as better places for career opportunities	P-RE-M, SOP, Fin
			-Non-local employees perceive promotion as unlikely for them in local public organisations	P-RE-M, Dis-Log, Fin
	Recruitment		-New international companies arriving in region offer better paid positions	Fin
Push	Salary	Dominant	-Employees leave to earn up to four times higher salaries in private sector, or start their own business	Fin
			-Low public sector salaries are even less attractive because of payment difficulties	Dis-Log, Fin
			-Complementary salary (e.g. moonlighting) can prevent turnover, but opportunities are scarce in the Afar Region	P-RE-M
			-Overtime work is perceived as excessively long and poorly paid in the Afar Region (potentially because of understaffing)	Dis-Log, Fin
	Facilities	Dominant	-Employees leave because of low health service standards, which they perceive to be better at NGOs and private clinics	SOP
			-Employees feel frustrated and get emotionally drained by not being able to provide health services due to lack of equipment & medicine	SOP, Dis-Log
			-Employees unlearn/lose skills because of lack of equipment.	SOP
			-Exposure to chemicals, unhygienic conditions, for employees, technicians and patients (P-HS)	SOP
	Location	Dominant	-Public sector workers seek locations with good moonlighting opportunities to supplement low base salary	P-RE-M, Fin
			-Employees and their families dislike the harsh climate and dull social infrastructure	P-RE-M
			-Lack of good schools and health services for family	P-RE-M
			-Professional and social life is complicated by language difficulties	P-RE-M
			-Cultural differences and distances inhibit bonding with patients, colleagues, community	P-RE-M
			-Perceived discrimination and unfairness by local management	Dis-Log
	Personal health and safety		-Aggression by patients because of poor services (resulting from poor facilities)	SOP
			-Exposure to chemicals, unhygienic conditions	SOP
			-Violence by management	Dis-Log
			-Poor access to health services for employees and family	P-RE-M, Fin
	Workload		-Emotional burden to not being able to provide health service due to poor facilities	SOP
			-Cascading effect when others leave as workload increases for the remaining workforce resulting in long, poorly paid overtime	Dis-Log, Fin
			-Perceived workload may be higher because of lack of control and autonomy	Dis-Log, SOP
	Management support	Dominant	-Demotivation through management’s lack of interest in the well-being of the primary process employee	Dis-Log
			-Personal relationships may outweigh professional ability in hiring/promotion	Dis-Log
			-Little recognition of professional abilities and autonomy	Dis-Log
			-Employees leave because they feel badly treated, sometimes even violently.	Dis-Log
			-Good management support drives retention	Dis-Log

*Abbreviations* of mechanisms (column 5): *P-RE-M* lack of social and personal opportunities valued by the healthcare employees, *Dis-log* dissonance between management logic and professional logic, *SOP* standards of service operations are hard to accept, *Fin* lack of financial improvement opportunities

### Step 4: Assessment and interpretation of the analysis

The iterative process to generate and identify mechanisms that are supported by the collected data and relate to existing scientific evidence and theory resulted in four mechanisms to explain the perceived turnover intentions among healthcare professionals in the Afar Region.

#### Lack of social and personal opportunities valued by healthcare employees (P-RE-M)

Clearly, our data imply a misfit between person and regional environment (P-RE-M, as inventoried in Table 1). Respondents indicate that the remote Afar Region offers poor perspectives for personal development, social life, and quality of family life. The climate is tough, the region is struggling to provide a reliable supply of water and electricity, there are very few leisure opportunities, the quality of schools is considered poor, and both local language and culture are different from the languages and cultures of the majority of employees. As the region is also considered expensive, the lack of moonlighting opportunities results in a misfit between the financial opportunities the healthcare professionals seek and the financial opportunities they find in the Afar Region.

At least until the opening of Samara University and School of Nursing, the perceived lack of educational development opportunities presented a misfit between personal development needs and the regional educational offering. The same holds true for career opportunities, which are perceived to be much better elsewhere, specifically in larger Ethiopian cities. While the two-factor theory classifies these factors as pull factors, the experienced lack of fit by respondents would cause them to be considered a push factors in the person-environment fit theory.

#### Dissonance between management logic and professional logic (Dis-Log)

Cultural differences may also drive the perceived lack of fit with management. The data indicate, however, that this lack of fit has other origins as well. Various professionals report that their professional logic—which emphasises quality of care, recognition of professional identity and autonomy—is hard to reconcile with the management logic. We propose the term dissonance to express that the difference in logic is not simply about rational priority setting. From the employee perspective, it seems rooted in management values and actions which conflict with their professional values and identity. While the literature suggests that various logics can co-exist in health service organisations, our data reveal that employees feel that the lack of recognition, respect and autonomy causes standing practices to result in dissonance with their professional identity.

The most vivid and direct illustrations of this turnover mechanism are the accounts of the use of violence by managers, and the unwillingness of management to enter the employee workspace because of bad smells. In addition, employees feel that professional competence is not considered in the case of promotion, hiring or selecting staff for education. Professionals also perceive that management does not share their priority for improving the quality of care for the patients; management is perceived to prioritise financial issues instead, often favouring locals over the majority of non-local employees. Employees feel unsupported in their continuous hard struggle to provide good care under high, draining workloads.

#### Standards of service operations are hard to accept (SOP)

The resource limitations, both in terms of facilities (rooms, ambulances) and medical equipment, cause employees to be disappointed about the services they can provide, lead to occupational hazards and cause them to lose professional competences because of lack of practice. Moreover, it is emotionally difficult not to be able to give patients the required services and have to watch patients suffer the (fatal) consequences. Altogether, these operating conditions can be considered so poor that despite the professional values to serve patients, they push employees to find alternative employers, where the operating conditions are acceptable for their professional values. There is also a misfit with personal values. The typically young employees strive to practice what they have learned and develop themselves, rather than lose their skills due to poor operating conditions.

#### Lack of financial improvement opportunities (Fin)

While public sector salaries in the Afar Region are not lower than elsewhere in Ethiopia, they may be less favourable in practice because of payment difficulties. Provincial budgets and treasury apparently cause problems for management to pay due salaries consistently on time. At the same time, employees indicate finding the Afar Region expensive and already have problems with the present salary. They are therefore open to opportunities to improve their financial conditions. One alternative is moonlighting; paid work in the private sector after office hours. There are, however, few private facilities in the region where moonlighting is possible, and their present jobs already demand long overtime hours. Alternatively, there are some opportunities to move to NGOs, start their own private facilities, or work for multinationals entering the region. In many cases, however, financial mechanisms will cause employees to move to other regions where the cost of living is lower and/or moonlighting opportunities are better. Thus, while this financial mechanism mostly pulls employees to other regions, some are pulled to non-public organisations inside the Afar Region.

## Discussion

Our findings reveal that staff turnover hinders the needed advancement of the public health system in the Afar Region. Our realist case study resulted in middle range theory in the form of four newly identified mechanisms that explain how the context formed by the Afar Region and the organisational practices in its health system cause so many of the healthcare workforce to have turnover intentions.

The first identified mechanism—lack of personal and social opportunities—relates to the remote rural context in general and is difficult to address within the realm of the health system. Let it be noted, however, that the contextual factor apparently influences local and non-local employees differently (‘for whom’ [23]). We therefore recommend to distinguish these two employee segments when further analysing the contextual mechanisms and when taking corresponding measures to improve retention. It may well be that the healthcare workforce built by the recently initiated local university is less likely to be pulled towards employer organisations in other regions. Similar interventions to develop a locally embedded workforce are worthy to explore in other remote sub-Saharan regions as well.

In contrast to the first mechanism, the second mechanism—dissonance between management logic and professional logic—and the third mechanism—operating standards which are hard to accept—lie within the management sphere of influence.

The second identified mechanism strongly suggests that employee retention is likely to benefit from better recognition, respect, autonomy, and management support of professionals. It addresses the management values and calls for a more vital mode of co-existence of the management logic on the one hand—with all the pressing difficulties of a developing rural region—and the professional logic and personal values of a young professional workforce in a developing country on the other hand. Perhaps such a vital mode can be found by involving professionals in the management tasks and responsibilities, thus adopting hybrid professionalism [46]. This may take the form of dual management or promoting professionals to managers to stimulate the blending of both logics. Interventions to better align the logics might also target operational improvements, as the third mechanism explains that the professional employees have difficulties to accept the current operating standards.

The distinction of the second and third mechanism witness the composite nature of the push factor management support, which has not been previously reported in scientific literature 19,21]. In the context studied, management support regards cultural norms, values and behaviours as well as support to maintain professionally acceptable operating standards. Both of these two mechanisms may well come into play in other remote rural sub-Saharan African regions, when facing comparable difficulties to maintain operating standards and to adopt management practices and values which are well aligned with those of the professional healthcare workforce.

The fourth mechanism—lack of financial opportunities—partly relates to management practices (salaries and benefits) and partly relates to context, e.g. to private sector activities. Interventions to strengthen public-private interactions in the Afar Region may be instrumental to improving these financial opportunities and hence to reducing employee turnover intentions. The importance of this mechanism may vary among remote rural regions in sub-Saharan Africa, as is the case for the effectiveness of corresponding interventions.

Our Afar Region case study findings confirm recent literature which emphasises the relevance of environmental and organisational factors to explain turnover intentions of healthcare employees (instead of prioritising individual attitudinal and behavioural factors) [21]. Moreover, rather than identifying context specific factors, the realist approach resulted in middle range theory in the form of four mechanisms and enabled to suggest practical improvement interventions which may well apply to other remote sub-Saharan regions.

We should not expect however that one-shot implementation of a set of interventions will directly result in substantial progress. Instead, considerable time and an iterative development approach are likely to be required [47]. We strongly encourage such a longer term approach and corresponding research to advance understanding of implementation of interventions to reduce turnover in the Afar Region, and in remote rural sub-Saharan African regions more generally.

A first limitation of our study is formed by the travel restrictions which inhibited us from visiting all administrative zones of the Afar Region. Hence, we may have missed some factors and mechanisms, and our study cannot claim to be complete.

Unfortunately, quantitative data were not available at the time of data collection and appeared difficult to collect reliable in retrospect. Data triangulation through the addition of quantitative data has therefore not been possible. It would have been particularly beneficial to have measurements of actual turnover and destinations (see also the ‘Background’ section). Further research on actual turnover is also called for as the relationship between turnover and turnover intentions may be intermediated by contextual factors [20].

## Conclusions

The shortage of healthcare employees working in sub-Saharan Africa, which carries sub-Saharan Africa carries over 24% of the global burden of disease yet employs only 3% of the global health workforce, forms a health human resources crisis. It is considered as a main obstacle to accomplishing health-related Sustainable Development Goals, especially in remote rural areas. In these areas, the resulting challenges are further exemplified by healthcare workforce turnover. Our realist case study in the Afar Region reveals four mechanisms that drive turnover intentions. In part, these mechanisms are rooted in contextual, regional, factors beyond the control of health system management. Other mechanisms, however, closely relate to management values and behaviours, especially in recognition and support of the professional values of the healthcare workforce. Our results therefore suggest that interventions to improve alignment of management values with professional values, and more generally, to improve management support for the professional workforce can reduce turnover. We strongly recommend future experimental research in this area.

## Abbreviations

Dis-Log: Dissonance between management logic and professional logic
FGD: Focus group discussion
Fin: Lack of financial improvement opportunities
HIV/AIDS: Human immunodeficiency virus/acquired immunodeficiency syndrome
P-RE-M: Lack of social and personal opportunities valued by the healthcare employees
SOP: Standards of service operations are hard to accept
